# Psychometric properties and validation of the polish version of the 12-item WHODAS 2.0

**DOI:** 10.1186/s12889-020-09305-0

**Published:** 2020-08-05

**Authors:** Agnieszka Ćwirlej-Sozańska, Bernard Sozański, Hubert Kotarski, Anna Wilmowska-Pietruszyńska, Agnieszka Wiśniowska-Szurlej

**Affiliations:** 1 Institute of Health Sciences, Medical College of Rzeszow University, Aleja Rejtana 16c, 35-959 Rzeszow, Poland; 2 Institute of Sociology, Social Sciences College of Rzeszow University, Aleja Rejtana 16c, 35-959 Rzeszow, Poland; 3 Faculty of Medicine, Lazarski University, Świeradowska Street 43, 02-662 Warsaw, Poland

## Abstract

**Background:**

The assessment of disability in a population is an important part of public health management. In this article, we examine the psychometric properties and validation of the Polish version of the 12-item World Health Organization Disability Assessment Schedule 2.0. (12-item WHODAS 2.0).

**Methods:**

A systematic random sample comprised 584 adult urban residents. The Polish version of the 12-item WHODAS 2.0 and the World Health Organization Quality of Life-BREF, Short Form (WHOQOL-BREF) questionnaire were used to assess disability and quality of life, respectively. Basic sociodemographic data and selected health-related data (e.g., pain and depressive moods) were also collected.

**Results:**

Good scale score reliability for the entire tool was confirmed in the study population (Cronbach’s α = 0.90; Composite reliability = 0.95). In confirmatory factor analysis (CFA), satisfactory values of the fit indices were obtained (comparative fit index, CFI = 0.999; Tucker-Lewis Index, TLI = 0.999; root mean square error of approximation, RMSEA = 0.004; standardized root mean square residual, SRMR = 0.043, *p* = 0.454). Good consistency was noted over time (correlation coefficient = 0.88). The tool was found to have an appropriate level of validity.

**Conclusions:**

We found that the 12-item WHODAS is short and easy to use, and it is suitable for use in the form of an interview during screening tests. This tool is appropriate for measuring the health status, functioning, and disability of an average population. It may be more relevant for studying populations with health problems. The 12-item WHODAS can be used to successfully obtain information about the general level of disability in a population.

## Background

Measuring the overall levels of disability and functioning in a society is becoming an increasingly important issue for developing public health priorities and for assessing the effectiveness of health interventions in different populations [1]. The World Health Organization (WHO) recommends that disability and functioning measures should be conceptually and operationally linked to the International Classification of Functioning Disability and Health (ICF) framework [2]. Therefore, the WHO has developed the World Health Organization Disability Assessment Schedule 2.0 (WHODAS 2.0) [3]. It is a standardized tool that can be used for population studies and in clinical practice [3]. The WHODAS 2.0 is available in 12-item, 36-item and 12 + 24-item versions [1, 2]. The shortened version, i.e., the 12-item questionnaire, is recommended by the WHO to use for brief assessments of overall functioning in surveys or health-outcome studies in situations where time constraints do not allow for application of the longer version [3] or where there is a need to use short standardized tools to study a large group of people to assess the occurrence of health-related conditions and determine factors affecting the occurrence of disability. Applying abbreviated versions of research tools with appropriate psychometric features reduces the cost of population research and allows researchers to draw reliable conclusions. The WHODAS 2.0 has already been translated into more than 47 languages, which indicates the need for a standardized measurement of disability in an intercultural context [4].

Several studies have confirmed the acceptable psychometric properties of the 12-item version of the WHODAS. Andrews et al. carried out confirmatory factor analysis (CFA) of the tool, and they indicated its good fit (Tucker-Lewis Index, TLI = 0.99; comparative fit index, CFI = 1; standardized root mean square residual, SRMR = 0.07; root mean square error of approximation, RMSEA = 0.04) [5]. Snell et al. showed the good reliability of the 12-item WHODAS (Cronbach’s α-coefficient of 0.92) [6]. Park et al. confirmed the acceptable reliability of the tool (Cronbach’s α-coefficient of 0.86) and conducted a CFA that indicated a good fit (TLI = 0.96; CFI = 0.95; SRMR = 0.06; RMSEA = 0.06). All individual items exhibited a satisfactory correlation with the WHODAS 2.0 summary score (0.42–0.67 for all 12 items), and the items were statistically significantly correlated with the relevant WHOQOL-BREF subscales, indicating the convergent validity of the 12-item WHODAS [7]. In Poland, a psychometric analysis of the 12-item WHODAS 2.0 has not yet been carried out.

With reference to the implementation of the ICF framework in Poland, the WHODAS 2.0 has been translated in accordance with WHO recommendations, and actions have been taken to assess the psychometric properties of this tool. The purpose of our work here is to assess the psychometric properties and validation of the Polish 12-item version of the WHODAS 2.0 in the study of an adult population living in the voivodeship city of Rzeszow in Poland.

## Methods

### Participants

The study was conducted in 2017 on a representative sample of adult residents of Rzeszow. The participants were selected from the population registry database made available by the Rzeszow City Hall. According to the data provided by City Hall, approximately 116,000 adults live in the city [8]. The sample size was calculated using the Online Sample Size Calculator (NETSTEL Software), assuming a 95% confidence interval and a margin of error of 4%. It was determined that the total planned number of surveyed people should be *N* = 597. To ensure the representativeness of the studied sample, systematic random sampling was used with a hidden division into layers [9]. The frame was ordered according to gender, housing estate, and age. Owing to this arrangement of the sampling frame, it was also possible to appropriately select additional participants if a person in the original sample refused to participate in the study. Each person who refused to participate was replaced by another person of the same sex living in the same estate and of a similar age. Fewer than 30% of people in the original sample were replaced. After checking the completeness of the collected data, 13 questionnaires with missing data were rejected. Finally, 584 complete questionnaires were included in the analysis.

### Procedure

The study was carried out using a direct interview, implementing a pen-and-paper interview method. The use of this technique enabled the collection of standardized information from a wide range of respondents. For inclusion in the study, participants must have been aged 18 or over, and informed consent was required. The study was conducted by properly prepared and trained interviewers in the respondents’ places of residence.

### Measures

The research tools were the 12-item WHODAS 2.0 and the WHOQOL-BREF questionnaire.

The 12-item WHODAS 2.0 was translated and adapted for use with Polish-speaking individuals by a team of experts from the council for the International Classification of Functioning, Disability and Health in Poland at the Center for Health Information Systems in Warsaw with the consent of the World Health Organization (WHO) [10]. The WHODAS 2.0 has been translated using the guidelines recommended by WHO and divided into five steps: 1 – forward translation; 2 – Expert panel; 3 – Back-translation; 4 – Pre-testing and cognitive interviewing and 5 – Final version [11].

#### The 12-item WHODAS 2.0

WHODAS 2.0 was developed on the basis of a comprehensive set of categories included in the ICF framework. It was designed as a general measure to assess health, functioning, and disability and can be used for many purposes, such as epidemiological studies. This questionnaire was researched by the WHO in many countries, where its reliability, convergent validity with other assessment instruments, and other psychometric properties were analysed, such as sensitivity to changes following an intervention and its relationships with health state assessments. The results of the field studies indicated a stable factor structure that can be duplicated across countries and population groups, one-dimensional domains, and good test–retest reliability [12].

The 12-item version of the WHODAS 2.0 is a shortened version of the 36-item WHODAS 2.0, and it is useful for performing brief assessments of overall functioning and disability in health studies when time limits do not allow researchers to use a longer version. The 12-item version explains 81% of the variance of the 36-item version [3]. It is available in three different forms; namely, a survey conducted by an interviewer, a self-reported measure, and an assessment by an authorized person. For this study, we decided to use a properly trained interviewer. The structure of the 12-item WHODAS 2.0 is designed in such a way that it contains two questions from each of the six areas of life included in the 36-item version:

Domain 1: Cognition (items 3 and 6 of the questionnaire);

Domain 2: Mobility (items 1 and 7);

Domain 3: Self-care (items 8 and 9);

Domain 4: Getting along (items 10 and 11);

Domain 5: Life activities (items 12 and 2);

Domain 6: Participation (items 4 and 5) [3].

Response options for each item ranged from 1 to 5 to indicate the level of difficulty or a problem, i.e., none (1 point), mild (2 points), moderate (3 points), severe (4 points), and extreme or cannot do (5 points). A simple method of calculating the results was used for the psychometric evaluation of the questionnaire [13]. The overall points for global disability therefore ranged from 12 (no disability) to 60 (complete disability), with higher results indicating a higher level of disability [3]. In addition, the individual scores in each domain were also calculated by adding up the results of the two relevant items [5, 14]. When only one item from the 12 items of the WHODAS 2.0 was missing, the average of the remaining 11 items was assigned to the missing item. If more than one item was missing, the survey was rejected [1].

#### The WHOQOL-BREF questionnaire

The WHOQOL-BREF questionnaire is a research tool designed to assess the quality of life of healthy and ill people for both research studies and clinical purposes. It allows for a homogeneous assessment of the quality of life of all respondents. It enables comparisons of the quality of life between different people and populations. The BREF (short) version of the WHOQOL questionnaire is based on the WHOQOL-100. The WHOQOL-BREF questionnaire enables researchers to examine quality of life in four areas:

Domain 1: Physical health, i.e., daily activities, dependence on medication and treatment, energy and fatigue, mobility, pain and discomfort, rest and sleep, and ability to work (7 questions);

Domain 2: Psychological health, i.e., appearance, negative and positive feelings, self-esteem, personal beliefs, thinking, learning, memory, and concentration (6 questions);

Domain 3: Social relationships, i.e., personal relationships, social support, and sexual activity (3 questions);

Domain 4: Environment, i.e., financial resources, physical and mental security, health and social care (e.g., accessibility and quality, home environment, opportunities to acquire new information and skills, opportunities and participation in recreation and leisure), physical environment (pollution, noise, traffic, climate), and transport (8 questions).

Responses were given on a five-point scale to indicate the level of difficulty or problems. The scoring was scaled in a positive direction, which means that a higher score indicated a higher the quality of life. The obtained domain results were transferred to a scale of 0–100 (where 0 means a very poor quality of life and 100 means a very good quality of life) [15].

The WHOQOL-BREF questionnaire was chosen for the convergent validity assessment because it was developed by the WHO and, like the WHODAS 2.0, is recommended for use in both healthy and nonhealthy populations [15]. The structure of this questionnaire is similar to that of the WHODAS 2.0 and is based on the ICF framework. The WHODAS 2.0 measures functioning, while the WHOQOL questionnaire measures subjective well-being. The WHOQOL-BREF questionnaire is most useful in epidemiological studies and clinical trials where quality of life is of interest [16]. In addition, the WHOQOL-BREF questionnaire was translated and tested for use in Poland according to the WHO’s international guidelines [17]. The psychometric properties of the WHOQOL-BREF questionnaire have been found to be acceptable by the WHO [18]. Suarez et al. observed acceptable construct validity, internal consistency, convergent validity, discriminant validity and concurrent validity using the WHOQOL-BREF questionnaire [19].

#### Sociodemographic data

Basic sociodemographic data (sex, age, and education) and selected health information were collected. The first question about the state of health concerned pain sensations in the previous 30 days (ICF b280, sensation of pain) and was measured using a Visual Analogue Scale (VAS). The second health question was related to emotional problems (ICF b152, emotional functions) in the last 30 days and was measured by the following question: “How often have you experienced depression, a depressed mood or sadness in the last 30 days?” The answers were ranked according to a 5-point Likert scale, where the options were “never”, “rarely”, “often”, “very often”, and “always”.

### Statistical analysis

The obtained data were analysed using StatSoft, Inc. programme. (2017), STATISTICA (data analysis software system) version 10, and the R software package, version 3.6.1.

### Factor structure

#### Confirmatory factor analysis (CFA)

The researchers conducted confirmatory factor analysis (CFA) to determine whether the standard 6-factor structure of the 12-item WHODAS 2.0 fit to the study population. To evaluate the model fit, Hu and Bentler two-index method was used [20]. The parameters were estimated using the diagonally weighted least squares method.

### Scale score reliability

#### Internal consistency reliability

Scale score reliability was assessed using Cronbach’s α-coefficient and Composite Reliability (CR) in the study population. The acceptable minimum for both measures is 0.7 [21–23].

#### Test–retest

Another measurement used to assess the reliability of the tool was repeatability assessment. It was evaluated using the test–retest method. The study was carried out on a group of 30 people, for whom two measurements of the 12-item WHODAS 2.0 were available. The average time between the two measurements by different interviewers was 6 days (range from 4 to 7 days). Test-retest reliability was analysed by means of the Wilcoxon test due to a non-normal distribution. The normality of the variable distribution was tested using the Shapiro–Wilk test. In addition, the reliability of the test–retest method was assessed by an interclass correlation coefficient (ICC) [24].

### Floor and ceiling effects

Floor and ceiling effects were calculated by determining the percentage of participants who had the lowest or highest possible results for the 12-item WHODAS 2.0.

### Validity

#### Convergent validity

Convergent validity was assessed by correlating the results of the WHODAS and the WHOQOL-BREF questionnaire, and the analysis was performed by examining Pearson’s correlation coefficient (when both distributions were normal) or Spearman’s test (when at least one distribution was not normal). We hypothesized that adults with a lower quality of life have a higher level of disability [19].

#### Known group validity

Due to the lack of a normal distribution, as assessed by the Shapiro-Wilk test, the Mann-Whitney test was used to assess known group validity of the 12-item WHODAS 2.0 using an external criterion. Moreover, we assessed the known group validity, which was based on significant differences in health, functioning, and disability between the distinguished groups. Based on the literature review, we selected two common health problems affecting the occurrence of disability in the adult population. We took into account the simplicity in assessing these problems and the possibility of assigning function problems to the ICF framework.

The first health problem was the occurrence of pain (ICF b280, sensation of pain). The pain level was assessed using the VAS scale. For the purposes of the analysis, a dichotomous variable was created to divide the studied population into the groups based on the following cut offs: VAS ≤ 4 and VAS ≥ 5. We hypothesized that adults with a higher level of pain are characterized by a higher level of disability [25].

The second considered problem was experiencing depressive feelings (ICF b152, emotional functions) in the last 30 days. The study population was divided into groups of people who do not or rarely experience (1 or 2 on the Likert scale) depressive moods and people who experience depressive moods often, very often, or always (3–5 on the Likert scale). We hypothesized that adults who experience depression more often have a higher degree of disability [26].

The analyses adopted a significance level of 0.05. Therefore, all *p*-values ​​below 0.05 were interpreted to indicate significant relationships.

## Results

In the studied adult population, there were 53.25% women and 46.75% men. The age structure was in line with the demographic trend in Poland and was distributed as follows: people aged 18–29 accounted for 22.60% of the respondents, those aged 30–44 accounted for 35.27% of the respondents, those aged 45–64 accounted for 30.14% of the respondents, and those aged 65 or more accounted for 11.99% of the respondents. Most of the respondents had higher education (43.66%) or secondary education (36.13%). The average level of pain on the VAS scale in the studied population was 3.15 points. Moreover, most of the respondents rarely experienced depressive moods (59.42%). According to the 12-item WHODAS 2.0, the average disability score for the study population was 18.55 ± 7.60 on a scale of 12 to 60 points. The average quality of life in the studied population was high at 73.48 ± 12.55 on a scale of 0–100 points. Regarding quality of life, the respondents rated the psychological domain (Do2) highest and the environmental domain (Do4) lowest (Table 1).

**Table 1:** General socio-demographic characteristics of the study population (*n* = 584)

Variables	Mean ± SD / n (%)
**1. Gender**	
Female	311 (53.25)
Male	273 (46.75)
**2. Age (years)**	
18–29	132 (22.60)
30–44	206 (35.27)
45–64	176 (30.14)
65 and more	70 (11.99)
**3. Education**	
Primary education	11 (1.88)
Vocational education	67 (11.47)
Secondary education	211 (36.13)
Higher education	255 (43.66)
Refusal to answer	40 (6.85)
**4. Pain (VAS)**	3.15 **±** 2.16
**5. Depressive moods**	
Never	145 (24.83)
Rarely	347 (59.42)
Often	66 (11.30)
Very often	16 (2.74)
Always	10 (1.71)
**6. Disability (WHODAS 2.0 version 12)**	18.55 **±** 7.60
**7. Quality of life (WHOQL-BREF)**	
Total quality of life	73.48 **±** 12.55
Do1 Physical health	74.74 **±** 16.18
Do2 Psychological	75.14 **±** 14.40
Do3 Social relationship	74.94 **±** 16.37
Do4 Environment	69.08 **±** 13.39

### Factor structure

#### Confirmatory factor analysis (CFA)

**Fig. 1:**
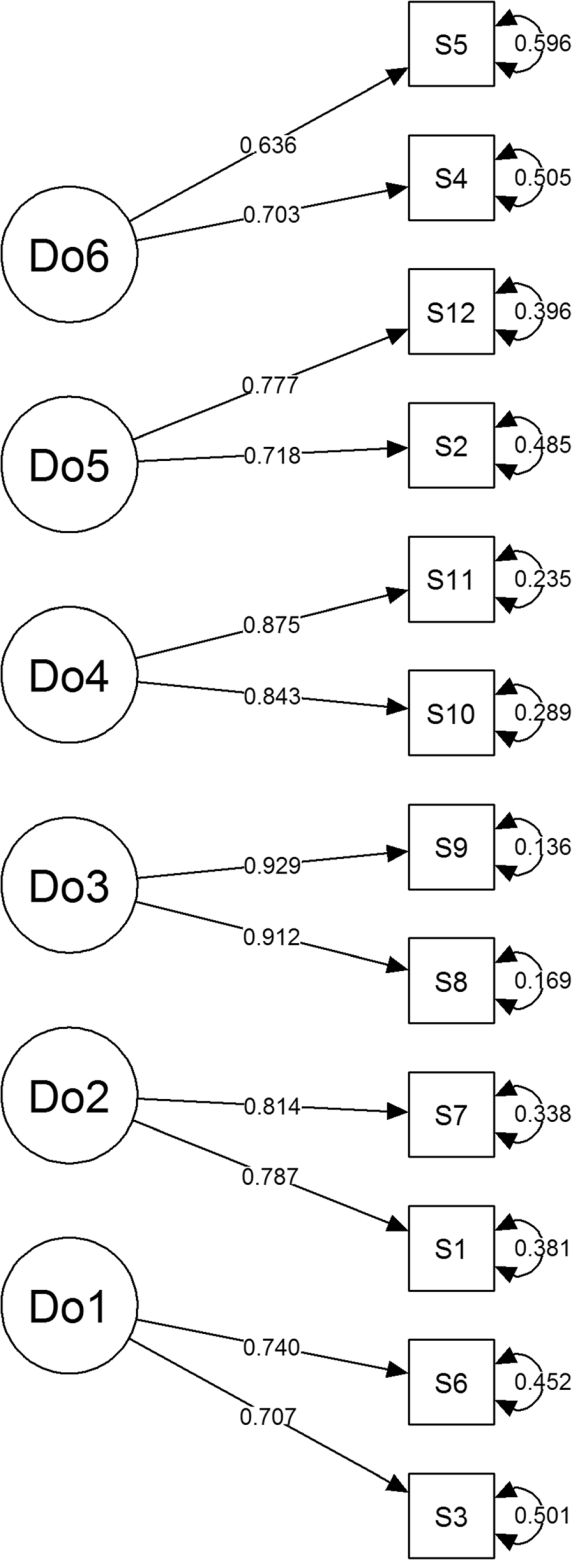
Confirmatory factor analysis of WHODAS 2.0 version 12

Confirmatory factor analysis (CFA) was performed using the diagonally weighted least squares method. The WHODAS has a six-factor structure (factors Do1–Do6). The loadings of individual items ranged from 0.636 to 0.929 and were statistically significant (*p* <  0.05), which means that all items were significantly correlated with the results of the corresponding subscale (Fig. 1).

Satisfactory values ​​of the fit indices (RMSEA, CFI, TLI, SRMR) were obtained for this structure. The six-factor structure was confirmed. The results indicated that a six-factor structure (understanding and communication; self-care; mobility; interpersonal relationships; work and household roles; and community and civic roles) of the WHODAS fits the data well, where CFI = 0.999, TLI = 0.999, RMSEA = 0.004, SRMR = 0.043, and *p* = 0.454.

### Scale score reliability

#### Internal consistency reliability

The scale score reliability of the entire tool for the study population was high. The Cronbach’s α test result for the whole scale was 0.90 and the CR – 0.95. The Cronbach’s α for individual domains ranged from 0.76 to 0.79. The CR for individual 2-item domains ranged from 0.62 to 0.92 (Table 2).

**Table 2:** Scale score reliability of the WHODAS 2.0 version 12 in the study population (n = 584)

WHODAS 2.0 version 12	Mean	SD	Min	Max	Cronbach’s α (95%CI)	CR
Total disability	18.55	7.60	12.00	50.00	0.90 (0.89–0.91)	0.95
Do1. Cognition	3.10	1.46	2.00	9.00	0.77 (0.74–0.80)	0.69
Do2. Mobility	3.30	1.79	2.00	10.00	0.76 (0.73–0.79)	0.78
Do3. Self-care	2.47	1.23	2.00	10.00	0.79 (0.76–0.81)	0.92
Do4. Getting along	2.99	1.59	2.00	10.00	0.80 (0.78–0.82)	0.85
Do5. Life activities	3.26	1.58	2.00	10.00	7.76 (8.60–6.98)	0.72
Do6. Participation	3.43	1.64	2.00	10.00	0.77 (0.74–0.80)	0.62

#### Test–retest

Consistency over time was examined by comparing the results of the first and second measurements in a group of 30 people. When using the Wilcoxon test to assess the changes between responses to over time, no significant differences were noted for the overall result or any of the domains. In addition, the reliability of the test-retest method was confirmed by the ICC. Time consistency ranged from very high (for Do2, ICC was 0.97) to weak (for Do3, ICC was 0.50). For the overall result, the ICC for the WHODAS was 0.91, which confirmed that the scale was consistent over time (Table 3).

**Table 3:** Statistical significance of changes between the test and retest studies (*n* = 30)

WHODAS 2.0 version 12	Test			Retest			***p***-value	ICC
	Mean	SD	Me	Mean	SD	Me		
Total disability	19.03	7.75	17.50	19.17	7.45	17.50	0.578^a^	0.91
Do1 Cognition	3.23	1.48	3.00	3.33	1.27	3.00	0.694^a^	0.61
Do2 Mobility	3.40	1.98	3.00	3.07	1.44	3.00	0.345^a^	0.97
Do3 Self-care	2.37	1.07	2.00	2.50	1.01	2.00	0.624^a^	0.50
Do4 Getting along	2.83	1.56	2.00	3.13	1.25	3.00	0.168^a^	0.63
Do5 Life activities	3.43	1.83	3.00	3.50	1.74	3.00	0.890^a^	0.89
Do6 Participation	3.77	2.14	3.00	3.93	1.91	3.00	0.433^a^	0.69

^a^ Wilcoxon test

### Floor and ceiling effects

The floor effect (an answer of “no problem”) value ranged from 49.83% (for item 5) to 84.93% (for item 8), while the ceiling effect (an answer of “extremely large” or “cannot do”) ranged from 0.17% (for item 3) to 2.05% (for item 7).

### Validity

#### Convergent validity

Convergent validity was tested by correlating the results obtained with the 12-item WHODAS 2.0 and the results of the WHOQOL-BREF questionnaire. Each domains of the 12-item WHODAS 2.0 were negatively correlated with each domain of the WHOQOL-BREF questionnaire; thus, a higher score on the WHODAS (higher disability) was associated with a lower score on the WHOQOL-BREF questionnaire (lower quality of life). All correlation coefficients were statistically significant at the level of *p* <  0.001. These findings confirm the hypothesis that adults with a lower quality of life are characterized by a higher level of disability (Table 4).

**Table 4:** The correlation of the WHODAS 2.0 version 12 and the WHOQOL-BREF (*n* = 584)

WHOQOL-BREF	WHODAS 2.0 version 12						
	Total disability	Do1 Cognition	Do2 Mobility	Do3 Self-care	Do4 Getting along	Do5 Life activities	Do6 Participation
**Total quality of life**	-0.54	-0.46	-0.46	-0.34	-0.41	-0.47	-0.48
**Do1 Physical health**	-0.66	-0.54	-0.64	-0.44	-0.43	-0.61	-0.55
**Do2 Psychological**	-0.38	-0.32	-0.32	-0.25	-0.30	-0.34	-0.35
**Do3 Social relationship**	-0.37	-0.33	-0.28	-0.24	-0.33	-0.29	-0.33
**Do4 Environment**	-0.36	-0.32	-0.29	-0.19	-0.29	-0.32	-0.33

All coefficients were statistically significant (*p* < 0.001)

#### Known group validity

We found significant differences in the health parameters that we analysed (e.g., depression and pain) between the selected groups. These findings confirm the hypotheses we put forward that adults with higher levels of pain are characterized by a higher level of disability, and people who experience depression more often have a higher level of disability. The results of the 12-item WHODAS 2.0 differed significantly between persons experiencing less and more pain and between people with less or more frequent depressive moods (Table 5).

**Table 5:** Known group validity of WHODAS 2.0 version 12

Groups		n	Mean	SD	Me	***p***-value
**At least frequent occurrence of depressive moods**	yes	92	24.70	23.50	9.65	< 0.001^b^
	no	492	17.40	15.00	6.56	
**Pain VAS ≥ 5**	yes	168	24.77	23.00	9.06	< 0.001^b^
	no	416	16.03	14.00	5.12	

^b^ Mann-Whitney test

## Discussion

To the best of our knowledge, this is the first study in which the psychometric properties and validation of the Polish version of the 12-item WHODAS 2.0 have been evaluated. This study is very important due to the need to implement valuable and reliable tools for assessing the state of health, functioning, and disability among the population, and this study enables the comparison of results between different groups, regions, and countries. It is also important in connection with the implementation of the ICF framework in Poland. In addition, the latest WHO resolution for the International Classification of Diseases 11th Revision (ICD-11) included the WHODAS 2.0 in the basic set of tools for the clinical assessment and screening of functioning and disability [27].

The results of our research have shown that the Polish version of the 12-item WHODAS 2.0 has good psychometric properties and can be useful for examining the adult population in Poland.

Our study has confirmed the well-fitting structure of the 6-factor tool. Very good values ​​of the fit indices (RMSEA (0.004), CFI (0.999), TLI (0.999) and SRMR (0.043)) were obtained for this structure. The loadings of individual items ranged from 0.636 to 0.929 and were statistically significant, which means that all items significantly correlated with the results of a given subscale. According to Hu and Bentler, a model fits well when the RMSEA is < 0.06, CFI and TLI > 0.95, and SRMR < 0.08 [20]. Similar results were obtained by Carlozzi et al. They confirmed the well-fitting factor structure of the 12-item WHODAS 2.0 by obtaining values ​​for individual tests, where RMSEA = 0.02, CFI = 0.99, and TLI = 0.99 [28]. Moreover, Andrews et al. examined a population of people aged 16–85 and obtained the following values ​​for the tests: RMSEA = 0.04, CFI = 1.00, TLI = 0.99, and SRMR = 0.07 [5]. Luciano et al. also obtained a good fitting factor structure [29]. The results confirming the six-factor structure of the 12-item WHODAS 2.0 are also consistent with other tests of the 36-item version [1, 30, 31].

We have confirmed the acceptable scale score reliability of the Polish version of the 12-item WHODAS 2.0 using the CR coefficient (for the whole scale was 0.95; domains ranged from 0.62 to 0.92). We found a very good scale score reliability of the entire Polish version of the 12-item WHODAS 2.0. The Cronbach’s α test value for the whole scale was 0.90. The Cronbach’s α value for individual domains ranged from 0.76–0.79. Cronbach’s α values ​​of 0.70 or more indicate adequate reliability [32]. Researchers using the 12-item WHODAS 2.0 in other languages have also confirmed the high values ​​of the Cronbach’s α test for this tool. Rehm et al., studying a cross-cultural sample from 19 countries, obtained a Cronbach’s α value of 0.86 [2]. Luciano et al. also found high internal reliability for the tool [29]. Abedzadeh-Kalahroudi et al. evaluated the psychometric properties of the Persian version of the 12-item WHODAS 2.0 among injured patients. They obtained a Cronbach’s α value for the whole tool of 0.91 [33]. In addition, Carlozzi et al., examining disability using the 12-item WHODAS 2.0 among people with Huntington’s disease, obtained a Cronbach’s α value for the entire tool of 0.94, and for individual domains, Cronbach’s α ranged from 0.74 to 0.90 [28]. Younus et al., studying the psychometric values ​​of the 12-item WHODAS 2.0 in patients with Kashin–Beck disease obtained Cronbach’s α values for individual domains ranging from 0.70 to 0.91 [34]. Axelsson et al. also confirmed the high scale score reliability of the 12-item WHODAS 2.0 in patients with anxiety and stress disorders (Cronbach’s α values were in the range of 0.83–0.92) [35]. Schiavolin et al., examining neurological patients by means of the 12-item WHODAS 2.0, obtained a Cronbach’s α value of 0.88 [36].

In our study, we confirmed the good repeatability of the 12-item WHODAS 2.0. No significant differences were noted for the overall result or any of the domains between the first assessment and the second assessment. It was confirmed that the Polish version of the tool was consistent over time (ICC value of 0.91). Younus et al. also confirmed the very good repeatability of the 12-item WHODAS 2.0 [34], and likewise, good consistency over time (1 week) was also affirmed by Moreira et al., who used the test–retest method and obtained an ICC of 0.77 [37]. Similar test–retest values (ICC value of 0.88) were obtained by Marom et al. [38].

In our study, we found a significant floor effect, while the ceiling was close to zero because the study was a cross-sectional study of a society rather than a clinical group. Despite the cross-sectional analysis of society without identified specific health problems, the tool can detect unobvious functional limitations. Katajapuu et al. affirmed a similar floor effect from 15 to 79% and no ceiling effect in a population of 2000 people with chronic musculoskeletal pain associated with mild or no disability [39]. Regarding several previous studies, floor effects were also detected for the WHODAS 2.0 domains or the overall score [4, 40].

We have also tested the convergent validity of the 12-item WHODAS 2.0. Each domain of the 12-item WHODAS 2.0 was negatively correlated with each domain of the WHOQOL-BREF questionnaire; hence, a higher score on the WHODAS (more severe disability) was associated with a lower score on the WHOQOL-BREF questionnaire (lower quality of life) (correlation coefficients *p* <  0.001). The good convergent validity of this tool was also confirmed by other authors. Tazaki et al. found significant correlations between the 12-item WHODAS 2.0 and the results of the WHOQOL-BREF questionnaire (p <  0.001) [41]. Luciano et al. found evidence for the convergent validity of the 12-item WHODAS 2.0 with the results of another tool assessing quality of life, i.e., the EuroQoL-5D questionnaire (EQ-5D) [28]. In addition, Schiavolin et al. also found evidence for the convergent validity of the 12-item WHODAS 2.0, obtaining moderate correlations with instruments for assessing quality of life [36]. This finding is in line with previous studies that showed a significant relationship between disability measured by the 36-item WHODAS 2.0 and quality of life measured by the Short-Form Health Survey (SF-36) [42, 43].

We have confirmed that the WHODAS 2.0 has satisfactory validity for people with different health statuses. In our study, the results of the WHODAS differed significantly between people experiencing less and more pain and between persons with less or more frequent depressive moods. Carlozzi et al. also observed significant differences in disability measured by the WHODAS between patients with milder and more severe forms of Huntington’s disease [28]. The ability of the 12-item WHODAS 2.0 to differentiate various health conditions was also confirmed by Schiavolin et al. The authors, using the Karnofsky performance status scale as a measure of general health, divided the study population into two groups: a group with no symptoms or mild symptoms and a group with active symptoms. The former group reported lower levels of disability than people with active symptoms [36]. Some previous studies have considered the group validity of the 36-item WHODAS 2.0. For example, Baron et al. divided patients with early arthritis into two subgroups according to the results of the Center for Epidemiological Studies Depression Scale and stated that the 36-item WHODAS 2.0 was able to distinguish patients with low and high depression symptoms [42].

### Limitations

The limitation of our research is the implementation of the tool among only urban residents. We plan to extend the research to the rural population. An additional limitation of our work is the use of a single question about depression instead of the standardized scale for validation analysis (known group validity). There were two reasons for this; it was necessary to limit the number of questions in the epidemiological study, and there were difficulties in choosing one short standardized scale for people aged 20–70. It should also be noted that two-item indicators of constructs are not optimal. For the purpose of psychometric evaluation, the research tools analysed the two-item domains indicated by WHO, but the practical conclusions based on the shortened version of the questionnaire should relate to the overall assessment of functioning and disability, not individual domains. The analysis indicates the proper selection of questions for the shortened tool by considering and properly representing all domains included in the 36-item WHODAS 2.0 version. If it is necessary to gain information about disability in individual domains, a longer version of the questionnaire should be used.

## Conclusion

In summary, we examined the psychometric properties of the 12-item WHODAS 2.0 in a study population of adult residents of the voivodeship city of Rzeszow in Poland. We found that the aforementioned disability assessment tool is short and easy-to-use, and it is suitable to use in the form of an interview during screening tests. The psychometric properties indicate that the 12-item WHODAS 2.0 is an appropriate tool for measuring the health status, functioning, and disability of an average population. This tool may be more relevant when studying populations with health problems. The 12-item WHODAS 2.0 can be used in place of the longer versions to successfully obtain information about the general level of disability. The implementation of the questionnaire recommended by the WHO enables researchers to conduct interpopulation statistical analysis and to more effectively manage health programmes in Poland, Europe, and the world.

## Abbreviations

WHODAS 2.0: World Health Organization Disability Assessment Schedule 2.0
WHOQOL-Bref: World Health Organization Quality Of Life, Short Form
ICF: International Classification of Functioning Disability and Health
WHO: World Health Organization.
CFA: Confirmatory Factor Analysis
CR: Composite Reliability
CFI: Comparative Fit Index
TLI: Tucker-Lewis Index
RMSEA: Root Mean Square Error of Approximation
SRMR: Standardized Root Mean Square Residual
ICC: Intraclass Correlation Coefficient
EQ-5D: the EuroQoL-5D questionnaire
SF-36: Short-Form Health Survey
